# Facilitating Constructive Discussions of Difficult Socio-Scientific Issues

**DOI:** 10.1128/jmbe.00153-21

**Published:** 2021-06-30

**Authors:** Gregory R. Goldsmith, Brenna M. G. Gormally, Rebecca M. Green, Aaron W. Harrison, Brian A. Hoover, Kenjiro W. Quides, Zachary Thammavongsy, Shana R. Welles, Bingjie Zhang, Kelsey M. Gray

**Affiliations:** a Grand Challenges Initiative, Schmid College of Science and Technology, Chapman University, Orange, California, USA

**Keywords:** conflict resolution, diversity, equity, facilitation, inclusion, reflective practice, STEM

## Abstract

Discussion can be an important and powerful tool in efforts to build a more diverse, equitable, and inclusive future for STEM (i.e., science, technology, engineering, and mathematics). However, facilitating discussions on difficult, complex, and often uncomfortable issues, like racism and sexism, can feel daunting. We outline a series of steps that can be used by educators to facilitate productive discussions that empower everyone to listen, contribute, learn, and ultimately act to transform STEM.

## INTRODUCTION

It should be no secret that science, technology, engineering, and mathematics (STEM) continue to have serious problems related to diversity, equity, and inclusion (1–5). Given the increased awareness surrounding issues such as racism (6, 7) and gender bias (8, 9), we have a renewed opportunity to make meaningful changes in STEM and to make it a place where everyone feels welcomed, valued, and supported in pathways to success. Many of us in STEM may be more open and eager than ever to talk about these issues in our classrooms and laboratories. What if we lack the tools to facilitate these discussions?

Discussing difficult issues is important and compelling. When we allow it to, it exposes us to new perspectives and leads to new ideas. It helps us recognize and investigate our biases and assumptions. It allows us to identify differences and commonalities. It encourages us to be better listeners and communicators. It promotes empathy and compassion. Discussion can be a powerful means of affecting change (10, 11).

What do we need to do to facilitate successful discussions? The approach in our undergraduate STEM education program is drawn from many resources (e.g., reference 12). We have applied discussion primarily to help our students explore issues ranging from gender bias in peer review to racial and socioeconomic disparities in COVID-19 (i.e., what may be referred to as socio-scientific issues [13]), but we describe our approach in a way intended to be broadly applicable. To see a specific example of a lesson plan, see Text S1 in the supplemental material.

## BEFORE THE DISCUSSION

We build a shared sense of purpose for the discussion and anchor the discussion in materials that bring evidence to bear on the topic. For instance, we may all agree that the purpose of the discussion is to understand how and why educational opportunities and attainment in STEM vary among different identities (1). We assign reading on the topic and ask students to provide brief written reflections about the materials before they arrive, so that everyone has time to consider the information and their own interpretations. This also provides the opportunity for everyone to contribute, even if they do not wish to speak out loud.

## STARTING THE DISCUSSION

At the start of discussion, we establish guidelines to foster community, build buy-in, and create a safe space. Common guidelines may include listening respectfully and without interruption, allowing everyone to speak who wishes to do so via an established method (e.g., raising their hand), asking questions for clarification and minimizing assumptions, focusing discussion on ideas rather than on the individuals bringing up the idea, and not asking anyone to speak on behalf of all people sharing a particular identity (10). We give everyone the explicit permission to make mistakes, give grace when they do so, and provide room to grow. These guidelines may seem evident, but we find that they can easily be forgotten amid a difficult conversation.

With a shared sense of purpose, established ground rules and materials that serve as the anchor for discussion, we often begin by asking everyone to consider a sentence completion exercise, such as “What struck me the most as I read/watched/listened to this was…” To reduce the hesitation to speak first, this can be coupled with a brief “think-pair-share” approach before returning to the whole group (14). We avoid beginning discussion with any form of summary or lecture, as this tends to shift the tone from participatory to didactic (Box 1).

## DURING THE DISCUSSION

When we facilitate, we are responsible for practicing careful listening, both to what people are saying and to the other cues they are providing through their choice of words, tone of voice, and their body language (15). We also hold primary responsibility for making sure that everyone feels their ideas have been given due attention. This can be as simple as repeating the idea back and asking for the person to confirm that you understood correctly, or thanking the person for being willing to share their idea. These methods maintain a space for all ideas, even if we do not agree with them. While facilitating, it is important to recognize that silence is OK and to resist the temptation to fill the void. Explicitly providing time to think can reduce monopoly of the discussion by a small number of individuals and amplify voices that may not otherwise be heard.

Ultimately, as facilitators, we strive to model the behavior we wish to see in those around us. We follow and enforce guidelines, listen carefully and ask thoughtful questions, keep conversation grounded in the materials, guard against misinformation, and ensure that everyone is heard.

## EFFECTIVELY ADDRESSING CONTROVERSY

How do we respond when controversy inevitably arises? When necessary, we return to the ground rules. For instance, if someone raises their voice and makes an accusation directed at an individual, we reply by reminding everyone in the room (rather than targeting an individual) that everyone agreed on a respectful dialogue focused on the ideas. We may also choose to explicitly address the emotions that we perceive to help everyone consider why they are experiencing those emotions. Remember that not all personalities and cultures will respond in the same way to conflict (16).

It is important to acknowledge the difference between a controversial comment and an offensive comment. Controversy can aid effective discussions (17). However, when someone says something particularly offensive, you may choose to pause the conversation. For instance, we say, “OK, let’s all pause for a second and get water or use the restroom.” This provides you with an opportunity to decide whether or not you believe that the conversation can continue in a constructive manner, which involves both your own comfort and ability, as well as the comfort of those around you. If an offensive statement has been made, then it may not be true that everyone will be ready and able to continue learning from one another at that time. If you decide to conclude the discussion, be sure to follow up with both the individuals directly involved and with the whole group to facilitate reflection.

If you choose to proceed with discussion following a controversial or offensive comment, you may ask everyone to write about what they are thinking and how they are feeling for a few minutes to increase the likelihood of additional constructive discussion. Before writing begins, ask clarifying questions and restate the issue at hand to address misunderstandings. For instance, we often observe strong reactions when people make statements that lack specificity (e.g., generalizations about a specific group). In deconstructing an overtly controversial statement, separate statements based on opinions and values from statements based on evidence. We may ask, “What evidence would we need to support this idea?” This forces us to confront underlying assumptions and can help reground the discussion. By framing the question to everyone, we avoid singling out a particular individual.

## AFTER THE DISCUSSION

At the conclusion of the discussion, we encourage everyone to reflect on what they have learned, what went well in the discussion, and what could be improved in the future. We try to do so both in the group setting, as well as individually. This can be as simple as having everyone write down three takeaway messages on notecards that you summarize in a follow-up email (18). We also make a plan for the next steps (e.g., further discussion or a specific action) and reach out to those individuals who we think may have been particularly affected with additional resources.

Like all of the skills we learn in STEM, learning to effectively facilitate difficult discussions will not happen overnight. It comes with practice. It comes with the recognition that we cannot (and should not) control every last moment. And like most things in science, it will not always turn out the way we had hoped. However, if we aim to address issues of diversity, equity, and inclusion in STEM, then we need to create equitable and inclusive spaces for a diversity of ideas to be shared, supported, and ultimately acted upon.

BOX 1

A collection of helpful phrases that can be used while facilitating discussions. Wherever possible, it may be useful to direct these to everyone participating in the discussion, rather than singling out an individual.

• What part of the materials did you find most confusing?
• Why do we think that this topic is controversial?
• What information do we have to support that idea?
• Can you restate that in another way so that we are sure we understand?
• What makes this hard to discuss?
• What do you imagine would be different today if…?
• What is your most important takeaway message from today’s discussion?
• What do we need to read about or discuss next time to advance our understanding?
