# Supplementary material for: Facilitating Constructive Discussions of Difficult Socio-Scientific Issues
Source: J Microbiol Biol Educ. 2021 Jun 30;22(2):e00153-21. doi: 10.1128/jmbe.00153-21 (PMC8442025; doi:10.1128/jmbe.00153-21)
Supplement: SUPPLEMENTAL FILE 1 — Download JMBE00153-21_Supp_1_seq2.docx, DOCX file, 0.02 MB [file jmbe00153-21_supp_1_seq2.docx]

**Appendix 1**

*Facilitating constructive discussions on difficult socio-scientific issues*

**Example Lesson Plan**

**Learning Objectives**

The following lesson plan was designed to facilitate a constructive discussion on issues of racial and ethnic disparities in health and healthcare in the context of the COVID-19 (SaRS-CoV-2) pandemic. We established the following learning objectives in line with the core competencies outlined in *Vision and Change in Undergraduate Biology Education*, as summarized in Clemmons et al. (2020):

1. Define health disparities (in the context of COVID-19).
2. Evaluate and interpret quantitative data demonstrating racial/ethnic disparities in health and healthcare, then identify the causes and conditions that contribute to COVID-19 heath disparities.
3. Differentiate between strategies for reducing COVID-19 health disparities from strategies for reducing overall population COVID-19 morbidity and mortality.
4. Consider how pseudo-scientific arguments can be used to support racist ideas and how the scientific community can address these issues.

**Student Population**

The lesson plan was carried out at a mid-sized, private, primarily undergraduate university. As of 2020, 52% of the students are White, 16.4% are Hispanic/Latino, 14.7% are Asian, 8.8% are two or more races, 2.6% are nonresident aliens, 2.6% are race/ethnicity unknown, 2.5% are Black/African American, 0.2% are Hawaiian/Pacific Islander, and 0.1% are American Indian/Alaska Native. In addition, 61% are women, 20% are first-generation to attend college, and 18% are awarded Pell grants.

Discussion of socio-scientific issues was conducted as part of a foundational seminar on critical thinking, problem solving, communication and teamwork in science and engineering. The participating students were first-year undergraduates primarily pursuing studies in STEM majors; however, there were always students from non-STEM majors in each class. The lesson plan was used in 10 different sections of the seminar where the average class size was approximately 25 students.

**Time**

Approximately 45-60 minutes.

**Pre-Discussion Reading Materials**

Students were asked to read the following articles prior to class and to look up terms that they do not understand:

1. Reeves TNF Sarah Reber, and Richard V. 2020. Race gaps in COVID-19 deaths are even bigger than they appear. *The Brookings Institution*. <https://www.brookings.edu/blog/up-front/2020/06/16/race-gaps-in-covid-19-deaths-are-even-bigger-than-they-appear/>
2. Chowkwanyun M, Reed AL. 2020. Racial Health Disparities and Covid-19 — Caution and Context. *N Engl J Med* 383:201–203. https://www.nejm.org/doi/full/10.1056/NEJMp2012910

**Pre-Class Discussion Post Prompts**

Students are asked to answer at least two of the following questions and/or add to other students’ observations on the online class discussion board.

1. What struck you about this reading and why?
2. What was the goal of this writing? Do you think they accomplished that goal?
3. What additional evidence do you think is needed to support the authors assertions?
4. What did you not understand, or confused you, and why?

**In-class Discussion Questions**

Students are asked to consider a selection of the following questions in both small group discussions and as an entire class.

*Define health disparities in the context of COVID-19.*

- What does BIPOC mean? What are benefits and concerns related to the use of this term?
- What are the differences between race and ethnicity?

*Evaluate and interpret quantitative data demonstrating racial/ethnic disparities in health and healthcare.*

- Why do the authors define a “crude death rate?”
  - *n.b.* Age-adjusting this crude death rate makes it such that relative to the proportion of the population from each race at a given age interval, the disparities are much higher than they appear at first glance.
- How did the Brookings Institute article choose which racial minorities to focus on?
  - *n.b.* Largest racial minorities compared to the majority of the population in the United States (white)

*Identify causes and conditions that contribute to COVID-19 heath disparities.*

- Based on the reading, is there evidence for biological or genetic differences in COVID-19 susceptibility based on race or ethnicity?
  - *n.b.* There are no biological or genetic differences in COVID-19 (unlikely that any will be identified, as compared to say, the occurrence of Tay-Sachs disease).
- How do socioeconomic status, chronic stress brought on by racial discrimination, and place-based risks contribute to racial health disparities?
- What experiences have you had regarding race/ethnicity and COVID-19?
- What search methods would you use to find accurate information on racial disparities in access to healthcare?

*Differentiate between strategies for reducing COVID-19 health disparities from strategies for reducing overall population COVID-19 morbidity and mortality.*

- How does biological variation among humans influence COVID-19 susceptibility, morbidity, and mortality? How can we acknowledge these differences to improve quality of life?
- Mortality rates for 18 to 24 year-old individuals remain very low. How does this age group catching and spreading COVID-19 potentially exacerbate the racial disparities in incidence and mortality?

*Consider how scientific arguments can be used to support racist ideas and how the scientific community can address these issues.*

- What other instances are you aware of where biology or genetics have been used to describe differences among racial or ethnic groups?
- Asian-Americans have been subject to a number of acts of hate due to the association of COVID-19 with its geographical origin of emergence from Wuhan, China. These associations are often linked to behavior (e.g., the consumption of wild meats from wet markets). How would you engage in conversation with someone who blames this ethnic group for COVID-19?

**In-class Synthesis Activity (*optional*)**

Look at this website: <https://covid19.emory.edu/>. Compare and contrast patterns within Orange County and Imperial County.

- 1. How do the total cases between these two counties compare?
  2. How do the demographics of the two counties compare?
  3. What differences between the two counties might be accounting for the differences in COVID cases?

**Formative Assessment, Metacognition, and Reflection**

Students are asked to conclude the class session by completing one of the following exercises (Angelo and Cross, 1993):

1. Minute paper – Students take one minute to write their main takeaways from the discussion.
2. Muddiest Point – In this variation of the minute paper, student focus their attention and writing on points from the discussion that raised the most questions for them or that they found to be most confusing. This can be especially helpful for discussions where students are likely to have questions they may not feel comfortable asking aloud.
3. Power Action – Students reflect on what they have learned and connect this with an action they will take as a result of what they have learned. For example, they may initiate a discussion with a friend who is not in the class, contact local or state representatives, or commit to practicing greater awareness of their own biases in daily life.

**References**

1. Angelo TA, Cross KP. (1993). Minute paper. *Classroom assessment techniques: A*

*handbook for college teachers*, 148-153.

1. Clemmons A, Timmons J, Herron J, Crowe A. *BioSkills Guide*. Core Competencies for Undergraduate Biology (Version 5.0). QUBES Educational Resources. [doi:10.25334/156H-T617](http://dx.doi.org/10.25334/156H-T617)
